# Gaming the system to care for patients: a focused ethnography in Norwegian public home care

**DOI:** 10.1186/s12913-019-3950-3

**Published:** 2019-02-14

**Authors:** Maria Strandås, Steen Wackerhausen, Terese Bondas

**Affiliations:** Nord University, Universitetsalleén 11, 8049 Bodø, Norway

**Keywords:** Home care services, Health service organization, Home care management, Nursing, New public management, Nurse patient relationship, Home nursing, Focused ethnography, Community care, Caring

## Abstract

**Background:**

With its emphasis on cost-reduction and external management, New Public Management emerged as the dominant healthcare policy in many Western countries. The ability to provide comprehensive and customized patient-care is challenged by the formalized, task-oriented organization of home-care services. The aim of this study is to gain deeper understanding of how nurses and the patients they care for, relate to and deal with the organizational systems they are subjected to in Norwegian home care.

**Methods:**

The focused ethnographic design is based on Roper and Shapira’s framework. Data collection consisted of participant observation with field notes and semi-structured interviews with ten nurses and eight patients from six home care areas located in two Norwegian municipalities.

**Results:**

Findings indicate cultural patterns regarding nurses’ somewhat disobedient behaviors and manipulations of the organizational systems that they perceive to be based on economic as opposed to caring values. Rigid organization makes it difficult to deviate from predefined tasks and adapt nursing to patients changing needs, and manipulating the system creates some ability to tailor nursing care. The nurses’ actions are founded on assumptions regarding what aspects of nursing are most important and essential to enhance patients’ health and ensure wellbeing – individualized care, nurse-patient relationships and caring – which they perceive to be devalued by New Public Management organization. Findings show that patients share nurses’ perceptions of what constitute high quality nursing, and they adjust their behavior to ease nurses’ work, and avoid placing demands on nurses. Findings were categorized into three main areas: “Rigid organizational systems complicating nursing care at the expense of caring for patients”, “Having the patient’s health and wellbeing at heart” and “Compensating for a flawed system”.

**Conclusions:**

Our findings indicate that, in many ways, the organizational system hampers provision of high-quality nursing, and that comprehensive care is provided in spite of - not because of - the system. The observed practices of nurses and patients are interpreted as ways of “gaming the system” for caring purposes, in order to ensure the best possible care for patients.

## Background

Norway’s public healthcare system provides healthcare to everyone in need, and is considered internationally to be a comprehensive and generous welfare model [1, 2]. Broad, ambitious welfare policy goals [3, 4] are costly, and Norway ranks among the highest of OECD nations in public spending per capita [5]. While the healthcare systems main task is to provide care for patients, it must also be financially sustainable [6], and the introduction of New Public Management (NPM) into Norwegian healthcare can be seen in conjunction with financial challenges in the public sector.

### New public management in home care

The term NPM is a common designation of various reforms and change-processes that have characterized public sectors worldwide. NPM has several different components including performance-measurements, fragmentation of services, contractualism, efficiency, decentralization, privatization, competition, governance and financial control [2, 7, 8]. The public care sector in many countries, including in Nordic countries, has been subjected to organizational reforms and regulations based on NPM principles [1, 2, 9]. Despite the goals of enhancing quality and reducing costs, evaluations after 30 years of NPM-reforms in the UK public sector conclude that NPM has been unsuccessful and rather led to higher costs and more complaints [10].

The effects of NPM-organization on nursing practice are significantly documented, and include: task-oriented and less flexible services, rationing of care, lack of nurse-continuity, increased reporting and documentation requirements, detailed goal-management, job-dissatisfaction and less time for: conversations, basic needs, caring, mental-health needs and nurse-patient relationships (NPR) [1, 11–18]. If the home care culture – meaning the ideas, values and rules guiding behavior and interaction [19] in home care – are under continuous pressure of efficiency and productivity, nurses may according to Martinsen [20] develop a more instrumental and standardized attitude towards patients who become at the risk of being objectivized. It is increasingly recognized that the organizational culture in healthcare institutions influence the quality of care provided [21].

### Organization of home care services

Since the implementation of The Coordination Reform [22] and the Healthcare service Act [4] in Norway, the responsibility for patients receiving long-term care has progressively shifted to municipalities [23]. Municipal health services encompass various kinds of institutional- and home-based care, with home-care nursing increasing most rapidly [23, 24]. Home-care patients vary in age, function, illnesses, and living conditions; although home care is in many ways more diverse than institutional-based care [24]*, the overall trends (in most Western countries) are aging patient groups with multi-morbidity and increasingly-complex needs* [25, 26]*. The reasons for an older and sicker home-care population are multifaceted.* Institutional closures, NPM’s perception of and implementation of rationalization of hospital- and nursing-home sectors have led to patients being discharged to their homes sooner [15, 23, 27]. Combined with demographic changes (aging populations) and a Norwegian welfare policy that encourages citizens to remain in their homes longer [28], the need for competent and experienced home care personnel is rising [29].

Norway has approximately 5.3 million inhabitants, and is divided into 422 municipalities ranging from just over 200 inhabitants to almost 675,000 [30]. Scattered populations challenge the provision of equal and comprehensive home care. As the responsibility for implementing efficient policies is pushed to the lowest level of the healthcare system, home-care areas are under great pressure to ensure effective service and manage service allocation within decided budget frameworks [31]. Community nurses have reported increased workloads and time pressures due to more and sicker patients, as well as increased patient flow between different services, resulting in additional administration and documentation. Consequently, working conditions and service provisions are affected, and nurses are pulled away from patient-centered care [15, 32–34]. These changes restrict nursing practice and shape the home-care culture.

Norwegian home-care services range from practical household assistance to advanced medical treatment, such as blood transfusions or stoma-care. Services are organized, managed and primarily financed by municipalities, and home care personnel include nurses, nurses’ aides and other formal caregivers [24]. Medical assistance is free for patients, but municipalities can charge for services such as practical assistance (e.g., washing dishes) *(*[35] *§8).* Patients in need of assistance to continue to live at home may apply to their municipality, and home care services are granted through statutory *decisions* that detail which tasks the patient will receive help with and how many hours/minutes per week will be assigned to perform these tasks *(4 §4–1,* [36] *§17,* [37] *§2–7,* [38]*)*. Similar to Holm et al. [39], we use the term *decision* to refer exclusively to this predefined and time-framed service-allocation.

Services are organized according to the assigned timeframe, reflecting only direct patient time and not time spent in driving, administration, documentation, interdisciplinary collaboration, etc. [38, 40], *activities that are often underestimated* [41]*.* Nurses work according to electronic worklists that detail which patients to visit each day, what tasks to perform, and how many minutes are assigned per task. Nurses are required to document all tasks they perform and to give reasons for any deviations from the worklist. Leaders may log into these electronic worklists and documentations to follow where nurses are and what they have done. Often, the timeframe assigned is insufficient, or patients need more time than the worklist implies, and studies show that nurses experience increased time pressure as a problem that hinders high quality home care [13, 33].

### Providing nursing care in a contemporary context

Nursing is a profession that is historically based on moral obligations towards the suffering human being. High-quality nursing depends on nurse-patient relationships (NPRs), which many consider fundamental to nursing [42–44] and which have health-enhancing potential [45]. Patients view NPRs as a way to develop meaning in their daily life in home care [46]. Caring sciences promote NPRs as essential, advocating that all caring is formed in relationships and stating the aim of relieving suffering and enhancing patients’ health and wellbeing [44, 47]. Even though healthcare personnel are obligated by law to provide professionally sound and compassionate services, less than 40% of community nurses report having enough time to safeguard patients’ needs for caring, social contact, and companionship [15]. Being unable to fulfill these obligations can cause stress, frustration, guilt, and loss of pride in giving good care [48]. Formalized and task-oriented services can lead to nurses’ professional judgement being overlooked in the healthcare organization, and NPRs, caring and individualized care may suffer as a result.

This manuscript is part of a larger ethnographic study examining NPRs in the NPM era [18, 45]. When collecting data in that study, the first author (MS) observed nurses and patients engaging in somewhat disobedient behaviors toward external management. Such disobedient behavior included e.g. not abiding by tasks and timeframes assigned in decisions, performing additional tasks, etc. In this article, we seek to understand and explain this witnessed phenomenon. Similar disobedient behaviors have been found among team leaders of home care areas, palliative care teams, nurses and residential care workers and managers in previous studies [9, 49–51], and have been interpreted as proof that employees do not comply with all predefined decisions [39]. This indicates that despite NPM influences, home-care cultures based on nursing- and caring values still exist [18].

Even though the overall mission of the healthcare system remains the same, the context within which services are organized and provided has changed markedly. This study adds contextual detail to the provision of contemporary home-care nursing, which may allow for increased understanding of the challenges faced by nurses and patients. To our knowledge, the phenomenon of disobedience towards external management remains unexamined from the perspectives of nurses and patients in home care.

## Methods

### Aims

To gain deeper understanding of how nurses and patients relate to and deal with the organizational systems they are subjected to in Norwegian home care. Our research questions are: What are nurses and patients perceptions of, and experiences with the organizational system in Norwegian home care? Which actions do they perform, and what are their understandings and justifications for their actions?

### Design

We chose an inductive and qualitative design, utilizing a focused ethnographic approach based on Roper and Shapira’s [52] framework. Focused ethnography enables exploration of a particular topic in a specific context, and is performed in subcultural groups rather than in entire societies [52, 53]. Data collection consisted of participant observation, field notes, and semi-structured interviews with nurses and patients, all conducted by the researcher (MS) over eight months in 2015/2016. Triangulating sources and methods increased dependability, as we could corroborate consistencies or divergences across data [54]. Focusing on caring through a cultural lens involves examining the meanings behind beliefs and behavior patterns (which may improve patient care) as they are shaped by the culture [19]. The term culture refers to patterns of behavior, symbols, values, beliefs, and knowledge that are created and taught through socialization-processes and eventually incorporated as common understandings that guide behavior [19, 55, 56].

The results reported in this manuscript originate from a previously published study by the same authors [18]. The two manuscripts convey the overall importance of the research. They address distinct and important questions and are not motivated around the same aim. Although the data material for both manuscripts were collected simultaneously from the same participants, they represents different topics of scientific interest and contribute new and different scientific knowledge.

### Participants and study setting

The study was performed in two Norwegian municipalities and included six home-care areas. Municipalities were selected based on geographical spread, practical feasibility and having a mixture of rural and urban areas to achieve variety while simultaneously identifying features in the field. The focus was on participants’ shared beliefs and practices; we worked under the assumption that they share cultural perspectives, even if they do not know each other [57]. A total of ten nurse-participants and eight patient-participants were included through purposive sampling for interviews [58].

Access to the field was gained by contacting each area’s nurse leader, who aided in recruiting nurse-participants. The prerequisite for participation was a minimum of one year home-care work experience. Through participant observations, potential patient-participants were identified. Before patients were invited to participate in interviews, the nurse-participants evaluated their health status, ability to provide informed consent, and ability and willingness to participate. Patients suffering from cognitive impairments were excluded. Nine patients were invited to participate in interviews, of whom eight agreed. Approx. an additional 120 patients were observed interacting with the ten nurse-participants, but these did not participate in interviews.

We strove for a sample of participants reflecting diversity in, age, experience in home care, education (nurses), and extent of health-service needs (patients) [52]. The intention was to increase the credibility of our findings within the examined municipalities/home-care areas and their transferability to municipalities/areas with similar organization of services. Nurse/patient characteristics were based on demographics of the home-care population [23]. Tables 1 and 2 shows characteristics of interview participants.

**Table 1:** Characteristics of nurse-participants

Gender	
Male	3
Female	7
Age	
Mean:	40,8 years
Spread:	32–55 years
Total work experience as a nurse	
Mean:	12 years
Spread:	2–32 years
Home care experience as a nurse	
Mean:	7,6 years
Spread:	1–15 years
Specialization^a^	
Diabetes	2
Dementia care and geriatric psychiatry	2
Intensive care	1
Palliative care	1
Wound care	1
None	4

^a^One nurse had two specializations

**Table 2:** Characteristics of patient-participants

Gender	
Male	3
Female	5
Age	
Mean:	77,6 years
Spread:	51–90 years
Home care experience	
Mean:	5 years
Spread:	1 month - 20+ years
Home care visits pr. day	
Mean:	3,25
Spread:	2–5
Visits/help from close family	
Daily	2
Weekly	4
Monthly	2

### Procedure

In line with a focused ethnographic approach [52, 53, 57], data collection began with participant observation during predetermined periods, meaning that the researcher was not fully immersed in participants’ lives. Observations were performed as part of the nurses’ everyday work and occurred at all times of the day, primarily in patients’ homes but also in the home care areas’ offices and while driving between patients’ homes. The durations of observations varied, but the researcher usually followed each nurse-participant through their workday, and nurse-patient interactions varied from 1 to 2 min to over an hour. (See Table 3 for frequency of data collection methods.) Observational data were recorded between visits to patients’ homes as hand-written field notes, focusing on the ways nurses and patients related to and dealt with organizational systems. Field notes included information about witnessed events (i.e. observational data), verbatim verbal exchanges, and the researcher’s personal reflections and interpretations of events. The researcher strove to be discreet and not to interfere when observing. Informal conversations were limited to times when participants were alone [55, 59], allowing the researcher to examine if interpretations of meanings behind observed behavior coincide with participants’ own understandings.

**Table 3:** Frequency of data collection methods

Observations	
Total time (hours)	135
Time range pr. home care area (hours)	8–45
Approx. no. of different patients observed, not invited to interviews	120
Nurse interviews	
Number of nurses included for interviews	10
Time range of interview duration (minutes)	44–75
Patient interviews	
Number of patients included for interviews	8
Time range of interview duration (minutes)	20–40

All participants were observed before interviews commenced. The goal of the semi-structured interviews was for participants to be able to expound on subjects they were concerned about, while also giving the researcher the opportunity to ask for elaborations about specific topics, explanations of observed events, and clarification of ambiguities. Thus, interviews provided insight into background meanings, values, concepts, and thought patterns (often shared on a cultural level) behind observed practices and articulated beliefs [19, 52].

Two interview guides were developed, one for patient-interviews and one for nurse-interviews. The interview-guides were originally developed for the previously published study [18], and exploring disobedient behavior was not the original goal of the interviews. However, some of the topics in the interview guides were relevant for the research focus of this manuscript, including experiences of nurse-patient interactions, experiences of everyday home care practices, and nurses were asked about their assessments, priorities, and time management. Semi-structured interviews allows for flexibility to explore emerging topics [60], and as the focus of this manuscript arose from observations done while collection data for the previously published study, many of the questions asked revolved around the observed practices of participants, to uncover the meanings behind the practices. Participants were encouraged to talk about situations they had experienced that stood out in positive and negative sense. Nurse-interviews were conducted either at the end of their shifts in a suitable room in the home-care offices or on a day off in their home. Patient-interviews were performed in their homes, according to patients’ preferences. Interviews lasted 30–60 min, and were audiotaped and transcribed verbatim by the researcher (MS). Data collection continued until saturation.

### Data analysis

Analysis was based on Roper and Shapira’s [52] framework for analyzing focused ethnographic data. The analysis-process began while data was collected [52, 57], and initially consisted of critical reflections on the field notes and interview transcriptions by the authors (MS, TB). The aim was developed on the basis of observational data from fieldwork related to the overarching aim of the larger study which was to explore NPRs in the NPM era. Verbatim transcriptions do not necessarily transfer what was said: participants’ words, facial expressions, tone of voice, and use of silence sometimes indicate that their responses are euphemisms for something else. To achieve interpretative validity, the researcher (MS) frequently checked interpretations with participants, thus moving back-and-forth between emic (inside view of nurse/patients) and etic (outside view of the researcher) perspectives in order to test perspectives against each other and gain deeper understandings of participants’ beliefs and practices [19, 57].

The researcher (MS) read all field notes and interview transcriptions several times, to gain an overall understanding of the content. The other authors (TB, SW) read samples of the field notes and interviews to obtain understanding. Data was organized using NVivo 11 [61]. Observational data and interviews with patients and nurses were analyzed and coded jointly. Focusing on answering the study’s aim, MS coded data by grouping sentences and text segments with similar content into meaningful categories (descriptive labels). Descriptive labels were examined and the content of each descriptive label was summarized to provide an overview and reduce the data to a manageable size. MS alternated between descriptive labels and summarized descriptive labels, while regularly reflecting on the interviews as a whole.

The analysis continued by organizing descriptive labels in order to identify patterns, focusing on emerging or persistent concepts and similarities/differences in nurses’ and patients’ behaviors and statements. Descriptive labels with similar content were sorted into *categories,* which were then sorted into smaller sets of *pattern-categories*. Through this process, we identified possible connections between information, and thus developed *themes* [52]. The co-authors checked the credibility of MS’s coding and contributed to analytical debate and validation of emerging themes. We continued the iterative process of coding, categorizing, and abstracting data, seeking to find patterns of behavior, beliefs, meanings and factors that influence the ways nurses and patients relate to and deal with the organizational systems they are subjected to.

Events and responses that deviated markedly from the rest of the data (outliers) were identified and used to test findings [52]. Participants were quite homogeneous in their articulated beliefs about NPRs and the importance of caring. Nurse-participants articulated beliefs about the organizational system were also fairly homogeneous. One nurse’s responses however, did not fit the patterns discovered in interviews with other participants, and did not correspond to observational data. The nurse was more positive towards the organizational system, but portrayed practices that were not in harmony with these responses. These responses were compared to other responses and observations to examine if we could find shared characteristics in the differences requiring closer examination, or if the responses represented true outlier beliefs.

To gain deeper understanding of the rich and complex data, we linked findings with existing literature. Insights, ideas and assumptions (memos) were written during the data collection and analysis processes to help assess if something needed further clarification [52]. The analysis-steps were not performed chronologically, as we moved back-and-forth between different steps, engaging in reflective/critical discussions about identified themes and patterns.

### Ethical considerations

The study conforms to the Declaration of Helsinki [62] and was approved by each home care area’s leader, The Regional Committee for Medical Research Ethics, and the Ombudsman for Privacy in Research at Norwegian Social Science Data Service. All participants received written and oral information about the study, including right to withdraw and guarantee of anonymity. Oral informed consent was obtained from patients who were observed but not invited for interviews; no personal data was collected from these patients. Written informed consent was obtained from participants included in interviews prior to their inclusion. Data was anonymized by removing names/locations and by changing details. Interview transcripts and audiotapes have been kept in locked files. This article is the work of three individuals, of which two (MS, TB) are nurses; all three have extensive experience with qualitative research.

## Results

The results focus on how nurses and patients relate to and deal with the organizational systems they are subjected to, and understanding the meanings behind their beliefs and practices. Observational and interview data was interpreted jointly to offer comprehensive understandings. Our interpretation of the data led us to categorize our findings into three main themes. For details on categories, pattern-categories and how the different perspectives contributed to the findings, see Table 4.

**Table 4:** Findings

Categories	Pattern-categories	Themes
Frustration and discontent due to organization and lack of time ^(O, P5, P6, N1–3, N6, N8, N9)^	Conflicting values	Rigid organizational systems complicating nursing care at the expense of caring for patients
Being managed by economic values ^(O, N1-N9)^		
Documenting just to be surveilled ^(O, N4, N6-N9)^		
Invisible “time-thieves” ^(O, N1–10)^	Trying to make ends meet	
Organization that complicate the provision of care ^(O, N1–4, N6, N8–10)^		
Unsolvable time-puzzles ^(O, N1–6, N8–10)^		
Organization at the expense of patients ^(O, P4, P6, P7, N1–4, N6, N8–10)^	Increased suffering related to care	
Time pressure affecting relationships, caring, quality and compassion ^(O, N1–6, N8–10)^		
Experiences of busyness and stress ^(O, P1–6 N1–6, N8–10)^		
Meaningful relationships and conversations ^(O, P1–8 N1–10)^	Understanding relationships as a fundament for nursing care	Having the patient’s health and wellbeing at heart
Being able to trust nurses causes feelings of safety and serenity ^(P2–8, N1–10)^		
Prioritizing conversations ^(O, N1–8, N10)^		
Nursing care tailored to unique and changing needs ^(O, P1–4, P6–8, N1-N8, N10)^	The value of caring actions, which cannot be measured	
Meaningful actions that cannot be fixed in decisions ^(O, P2, P3, P5–8, N1–10)^		
Focusing on the patient’s needs ^(N1–6, N8–10)^		
Nurses making autonomous choices based on professional judgement ^(O, N1–9)^	Professional autonomy	Compensating for a flawed system
Juggling minutes ^(O, N1–7, N10)^		
Manipulating timers and decisions ^(O, N1–4, N6–10)^		
Patients adjusting their behavior to ease nurses job ^(O, P1–5, P7, P8, N1–6, N8)^	Accepting and adjusting behavior	
Nurses surrendering to the system ^(N2, N3, N5–8)^		
Grateful patients with few demands ^(O, P1–8, N2, N3, N6)^		

Patients (P1–8), Nurses (N1–10) and Observational data (O)

### Rigid organizational systems complicating nursing care at the expense of caring for patients

Home-care organization is perceived by nurses as increasingly complex, and many factors influence nursing practices. Interview responses and observational data indicate that lack of time, rigid organization, and focus on profitability may affect NPRs and restrict nursing care, having unfortunate consequences for patients. Most nurses explain how they feel like their workday is governed by economic values rather than patient-oriented or caring values:

*We have to follow a system that is based on economic values. In my mind, that’s wrong because we are taught that other values are more important than economic values. We are forced to work towards something that’s very different from the values we learn in our profession and education. It’s a collision. I don’t mean we should waste resources, but we work with people. We work with life and death. (Nurse 8)*

Services are perceived by nurses to be suboptimal, because organization of care is based on what is profitable, not what patients need. Home-care areas receive financing from municipalities according to timeframes granted in predefined decisions. Nurses’ worklists are limited to this timeframe and details about which patients to visit, which tasks to perform, and how many minutes each task is assigned. If nurses spend less time than assigned, the municipality may reduce the patient’s allocated time, and the home-care area loses financing:

*The time must be correct according to decisions. The municipality is paid for the time assigned in decisions. If we use more time because we are nice and do the dishes or change a wound, or if the morning-care takes longer, we aren’t paid … Everything is based on economics. (Nurse 7)*

When entering patients’ homes, nurses start a timer on their phone which logs how many minutes are spent with the patient and whether it is in accordance with the given timeframe. Several nurses feel bounded by this timeframe, while others see it as an estimate. Leaders may track in real time where nurses are and what they are doing through this system, making some nurses feel surveilled and distrusted. One nurse however, felt that it was positive that leaders could track their actions, as potential slackers would be revealed.

Many nurses express that it is unnecessary that patients need predefined decisions on every little thing. They explain how they feel forced to abide by the worklists and not deviate from them. Help should be provided when help is needed, and predefined and time-controlled tasks reduces their ability to adjust care to patients’ changing needs:

*The route [patients to visit and tasks to perform] has been set for me and my role is basically to follow it. I cannot make the assessments myself. It’s very sad that I don’t get to evaluate how much time to spend somewhere. Needs vary from one day to the next. There may be days when they only need half the time they have decisions for, while other days they suddenly need double, and then some. (Nurse 1)*

Nurses explain how the clinical procedures they perform grow increasingly demanding and time-consuming as patients get older and sicker. They experience that only the sickest patients are assigned services, and that budget cuts force nurses to prioritize between basic needs to determine which needs are the most vital in any given situation:

*Those who receive help are those who need the most help. It is very harsh. Their needs are very detailed in the decisions. If there is anything they [the municipality] think patients don’t need help with, they won’t get it. (Nurse 4)*

Nurses seem to have many patients to care for, and nurses express that visits often must be adapted to their worklists rather than to the patients’ needs. Consequently, patients may be helped to bed at 7:30 PM and not helped up again until 11:30 AM the next day, or intervals between medications may be prolonged or shortened due to the nurse’s schedule. A few patients described events where nurses had canceled visits, such as administering insulin, because they did not have time.

According to many nurses, the chase to fulfill all tasks comes at the expense of compassion, caring, and quality. When time is limited, nurses do what is in decisions and nothing more. Although they may be able to swiftly perform physical tasks, psychosocial needs are more challenging and time-consuming, and when time is limited, nurses sometimes avoid initiating conversations:

*Decisions puts the breaks on that. Because sometime I don’t dare to ask [how the patient is doing]. I have no time to ask. I can see that something’s wrong, but I can’t ask because I don’t have the time to listen to their answer. (Nurse 6)*

Observations show that visits are often short: sometimes as many as five patients are visited within 20 min, including time for transportation and documentation. Fragmented visits occur when nurses cannot complete all tasks at once and must return later, or when visits are split between several caregivers. Since nurses are prioritized to perform invasive procedures and provide medication, they lack time for clinical observations, which may lead to signs of deterioration going undetected. Several patients express frustration over fragmented visits, unpredictability, lack of caregiver continuity, not receiving adequate help, and nurses not responding to their requests. Telephone-calls from other patients requesting help often interrupt caring encounters or procedures, and some patients believe that those interruptions and the short/fragmented visits demonstrate how busy nurses are. Patients appeared to experience such interruptions as more problematic than nurses, and many patients feel sorry for nurses and therefore avoid asking for all the help they need:

*I’m sure I would get help if I called. There’s not a doubt in my mind. They have so much to think about so I don’t ask. (Patient 4).*

Understaffing is articulated as another problem and nurses say that it is impossible to find time for all tasks they are required to perform. Meanwhile, observations identified variations regarding how busy nurses are. Although several days are quite demanding with high workloads and many patients, other days are calmer, often as a consequence of home care patients being hospitalized and therefore freeing up time on the nurses worklists. On demanding days, nurses may not finish all the tasks they are expected to perform, yet, nurses say that their leaders do not endorse working overtime due to the economy. When workdays are demanding, nurses therefore rush through visits, do tasks between visits, during breaks, at the end of shifts or postpone tasks:

*You can’t spend the time people are assigned because you know you don’t have enough time. Then, I’m punished because I may have stayed overtime because I used the time they were assigned. Any choice you make is wrong somehow (Nurse 1).*

While one nurse experienced the current organization of home care as satisfactory, most are blunt about their frustration and discontent; they refer to the organizational system as cumbersome, inefficient and a hurdle in the way of providing care. If nurses use less time than the patient is assigned, the decision-time may be reduced; thus, nurses stress over using enough time, while simultaneously ensuring that everyone receives help. Consequently, time becomes a focus:

*The nurse’s smartphone lies on the patient’s living room table with the screen on. The timer is visible, showing how many minutes have been spent and how many remain in the decision. This repeats all day long. The phone is always present. (Field note, day 7).*

Observational data show that nurses spend considerable time on organization-imposed administrative work, documentation, driving, interdisciplinary collaboration, and unforeseen events such as patients becoming ill, unexperienced colleagues making mistakes, or nurses forgetting equipment. These tasks are referred to as ‘invisible work’ by the nurses who explain that even though they are expected to document all tasks they perform, they choose only to document what they consider as important, stating that documentation steals time from patients.

### Having the patient’s health and wellbeing at heart

The fundamental drive behind most nurses’ efforts is expressed as a sense of joy and meaning in doing a good job, helping someone, mastering challenges, and making a difference. Nurses and patients highlight developing NPRs and caring for psychosocial needs as essential to enhancing health, preventing illnesses, and allowing patients to remain at home. Establishing trust and helping patients feel safe is described as fundamental. Nurses are particularly concerned with patients being able to trust them, stating the significance of conversations, responding to patients’ requests, and keeping their promises. Most patients explain how nurse interactions make them feel happier, calmer, and safer:

*It is so reassuring to talk with them. No doubt about it. I feel safer. If someone doesn’t feel safe … That must be awful … (Patient 7).*

There are no decisions that allocate time specifically for conversations, and nurses say that they deliberately initiate conversations while performing tasks to compensate for them being unable to sit down and have a proper conversation. Many patients also initiate conversations whenever they can, stating that they are used to nurses being in a hurry. For patients, the personal significance of NPRs and social interaction seems to vary. Some patients appear content with short visits and superficial conversations, while others need more. Nurses explain that they constantly evaluate how pressing a patient’s need for conversation is, and that they try to prioritize listening and talking with a patient when they sense that the patient truly needs it. According to several nurses, it is not about *having* the time for conversations but *taking* the time, often at the expense of other tasks. Using gestures like sitting down and taking off their jackets and shoes, nurses signal to patients that they are making time to listen to their concerns:

*The patient is supposed to shower, but doesn’t want to. He says that he dreads it. Says “I’m sick” and “You don’t understand what it’s like”. He wants us to come back later. The nurse explains that we can’t do that, it’s too much driving back-and-forth. She sits down, listens, and explains. Uses plenty of time persuading. (Field note, day 3).*

While patients are still alone after nurses leave, they express that they feel less lonely and stress the meaningfulness of even short conversations. Some patients express that they consider the practical tasks as avocations compared to nurses visiting them and talking with them:

*What matters the most is not the tasks they perform. It’s everything else. That they listen to me, take me seriously, and care about me. It makes me feel like I am worth something. (Patient 2)*

Observations show that all nurses perform numerous small but meaningful actions that appear to be difficult to detail in decisions. These caring actions may include: hugging, holding hands, listening to patients’ concerns, etc. They may also include practical tasks such as getting their mail, opening a window, or setting a patient’s hearing aids in place. Patients articulate how such tasks often cause them concern, and how significant it is that the nurses are helpful and understanding. Through caring actions, nurses attempt to tailor care according to patients’ individual and changing needs, wishes, and condition:

*We are working with sick people. No days are the same. Often, we get there and the patient is in bad shape. We want to nip it in the bud, go to the office and pick up equipment to take blood-tests so nothing can develop*. (Nurse 3)

Findings indicate that nurses’ and patients’ perceptions of what characterizes high-quality nursing differs from what is valued in decisions, and some nurses express that they are fighting the system to safeguard patients’ needs and wellbeing. Observations show that all nurses repeatedly choose to perform additional tasks, or tasks that deviate from, or were not granted in decisions. Nurses say that their loyalty lies with patients and that their focus is on identifying and safeguarding patients’ changing needs, not on fulfilling predefined tasks or keeping budgets:

*I’m there to do a good job for our patients … I try to help them with what they need. To many, that’s a good and dignified old age. Sometimes, that does not match the budget or timeframes, but I don’t think I’m really there to keep budgets for the municipality. That’s not what I’m employed for. I try to stay within the limits as much as possible, but, I have to do what’s right for the patient. I’m not an economist. (Nurse 5)*

Patients express that most nurses do their best to help them and are sensitive to their needs. Nurses regularly ask if patients need help with anything, behavior which some patients say make them feel like they are not inconveniencing nurses. Nurses say that they prioritize conversations, additional tasks and caring actions over breaks and administrative tasks, although it adds to their workload and stress:

*I go the extra mile while I’m with the patient. I feel it afterwards though. I get really, really stressed sometimes and feel that I probably have a pulse over 150. I think I hide it. (Nurse 3)*

### Compensating for a flawed system

Observations indicate that nurses and patients have developed different strategies to compensate for lack of time and for organizational challenges. Patients mostly appear to accept the reality and adjust their behavior accordingly, stating that they cannot expect to have all their needs met and have to respect that nurses cannot stay long:

*They [nurses] say for instance that some patients come first. You have to respect that. (Patient 3)*

Several patients articulate that they generally receive help when needed. Observations and interview responses indicate that most patients place few demands on nurses and are grateful for whatever help they receive:

*The patient is old and fragile, but seems grateful and content. “They are so nice” she says, “We are fine, you are doing your best.” It’s 4:30 PM and this patient is served her evening meal and medicine. Nobody is stopping by until tomorrow morning. (Field note, day 4)*

Many patients seem to trivialize their own pain and suffering and express compassion for other patients who they believe are worse off. Nurses are used to patients’ few demands, explaining:

*You have to remember what generation this is. Their generation was raised to save. They sewed clothes and exchanged it for food. They experienced war. They’re used to not asking for anything. (Nurse 6)*

Many patients that do not suffer from cognitive impairments appear to frequently adjust their behavior to ease nurses’ work. Patients express that when they have good days, they try to manage tasks on their own, not wanting to leave everything for nurses. Depending on their physical condition, patients perform various actions to help nurses. Some explain that they try to be cheerful and pleasant with nurses; others try to hurry or prepare equipment they know nurses will need to perform procedures:

*The same routine happen every time. The old couple jumps off the sofa the second we arrive. The patient walks slowly towards the kitchen, opening the buttons on his pants as he goes. His wife finds the insulin and the nurse adjusts the dosage. The patient enters the kitchen with his pants halfway down. He sits in his regular chair as the nurse injects the insulin swiftly and puts on his compression-socks. They talk and laugh for a few minutes before the nurse leaves. (Field note, day 15).*

Nurses appear less accepting of the organizational system, and state that individualized and holistic care is provided despite the organizational system, not because of it. A few nurses express a sense of wanting to give up and surrender to the system, feeling that they must accept that they cannot help everybody with everything. Such attitudes are less visible in their behaviors and most nurses seem to attempt to live up to their ideals and thus act more disobediently towards the system.

Nurse leaders’ interpretations and implementation of the organizational system seem to affect nurses’ behaviors. Some nurses state that their leaders view the assigned time as an estimate, leaving room for interpretations of the decisions, while other leaders follow decisions rigidly. Findings show that nurses also vary in how strictly they abide by organizational rules, predefined tasks and timeframes. Nurses seem to aspire to help patients while simultaneously adhering to the organizational rules, but all nurses in this study were observed portraying somewhat disobedient behavior with varying extent, frequency and type of actions. Some nurses follow the rules more strictly and appear more focused on the timeframes set, than other nurses who feel less obligation towards the organization.

Nurses frequently juggle minutes between patients - taking time from one patient and spending it on another - explaining that they are competent and able to evaluate how their time is best spent. Nurses feel that they know the patients best, and they want the freedom to help patients when they need help and to tailor nursing according to patients’ shifting and individual needs:

*I feel like this is forced on me, the timers and decision-times. I don’t like it because I want to spend my day … I can get all patients I’ll visit, but I am capable of assessing who needs more time and who needs less time. Who needs the most time - that I can assess! Use my professional education as a nurse, as a geriatric nurse. (Nurse 6)*

Many choose to perform tasks even if it conflict with the decisions, saying that they base choices on their own assessments and patients’ needs or wishes. Some do it in secret and document as if they follow decisions to the letter, but feeling like they are doing something illegal by not abiding by the decisions. Others are open about their assessments and defend choices to their leaders, adding that their reasons are mostly accepted by the leaders. Though having to defend their choices to leaders, nurses are not disciplined for their disobedient behaviors which can indicate that some leaders might share nurses perspectives. Nevertheless, many nurses express frustration over having to defend their choices at all, stating that leaders should have faith in their professional assessments.

The most widespread way of gaming the system is manipulating the timers to show them using the exact amount of time that is assigned. Many nurses start the timer before entering a patients house, let the timers keep running after they leave, or change the timers afterwards. This is to ensure that patients do not loose decision-time if for some reason nurses use less time with the patient. Many nurses feel disobedient and refer to themselves as rebels:

*Although there is no room for it, I’ll do it. I don’t care about that. I’m a little rebel. I don’t look at the clock first and say “no, I have to see if I have time.” I don’t care about that, I’ll just do it. I think of the patients as a whole, of everything. (Nurse 8)*

Some nurses explain that they manipulate decisions by overestimating how much time patients need, applying for additional services, sneaking in services camouflaged as something else or finding free alternatives for payed services. Nurses articulate that they do these things to ensure that they have enough time to maintain NPRs, care for patients’ psychosocial needs, and create wiggle-room to deal with unforeseen events. As patients grow older and sicker, nurses have to respond to more alarms, and the opportunities to shorten, skip, or rush visits become increasingly difficult:

*They say that if we’re understaffed we can skip what’s unnecessary. But what the heck isn’t necessary? When you administer medicine to everyone, who should you skip? If you arrive and they have decisions on insulin and personal hygiene, what are you going to say? “No, I’m only giving you insulin, but you won’t get help to go to the bathroom”? You cannot do that! I feel that we are a bunch of damn obedient people who really could refuse doing this, just to set an example. But we do everything anyway. Whom can we skip? (Nurse 2)*

Imbalance between caring needs and nursing resources leads to some low-priority tasks sometimes remaining undone or being postponed. The quote above illustrates the frustration expressed by many nurses.

## Discussion

Findings show that rigid organization, predefined tasks, high workload and time constraints limit nurses’ possibilities to tailor care to patients’ individual and changing needs, to customize the time of visits, and to act according to their own professional standards. It is evident that nurses’ beliefs, values, and ideals strongly influence their practices, and their understanding of NPRs and individualized care as fundamental remains strong. Findings indicate cultural patterns regarding nurses’ somewhat-disobedient behaviors and manipulations of the organizational systems that they perceive are based on economic rather than caring values. Similar forms of “culture clash” between NPM and nursing is documented in several healthcare settings [1, 6, 17, 49, 51, 63], and it is plausible that types of disobedient practices also could be found in other settings, by other healthcare personnel or leaders, and potentially taking different forms [9, 49, 51]. Furthermore, findings indicate that patients share nurses’ perceptions of what constitutes high-quality nursing. Patients adjust their behavior to ease nurses’ work, and they avoid placing demands on nurses when possible. These practices are interpreted as ways of “gaming the system” for caring purposes, in order to ensure the best possible care for patients within the current organizational system in Norway.

The identified conflicts between NPM and nursing may be understood in light of Habermas’s [64] concepts of “system world and lifeworld”. The system is characterized by strategic and instrumental goal-rationality rooted in and managed by the interests and powers of economic agents. Under this definition, NPM clearly originates and falls within the system. The lifeworld is the lived world of people and their relationships, it is the world of communication, of cultural practices, interpersonal values socially grounded understandings. From this perspective, the professional nursing practice originates and unfolds within the lifeworld. Both worlds are necessary in the modern society, but the system’s colonization and instrumental rationalization of the lifeworld is a primary concern of Habermas [64], and as this article shows, it is also a concern of nurses. NPM’s interference and attempts to “colonize” NPRs and nursing care, is an example of the system’s interference with the lifeworld [18, 64]. Nurses disobedient behaviors can be seen as an attempt to resist a “colonization” by NPM.

Nursing work comprises a significant portion of most operation budgets in home care, and nurses’ time is a fundamental resource in the healthcare system. Financial recourses will always be limited compared to the endless needs of patients, and the question is not whether nursing is rationed, but whether it is rationed well. While nurses seem to be required to prioritize medical, technical, and administrative tasks, nurses and patients highlight the equal importance of conversations, caring, NPRs, clinical observations and overall individualized care, in line with the caring science [44, 47]. Prioritization of resources is value-laden and nurses face moral dilemmas because nurses’/patients’ and NPM-organizations’ perceptions conflict as to what is most important in enhancing patients’ health and ensuring their wellbeing. Martinsen [20] refers to the focus on productivity and efficiency as a “cold-wave” and argues that patients are at risk of being objectivized and depersonalized. Meanwhile, research shows that willingness to care for elders remains despite worsening working conditions [65]. Nurses attend to the healthcare needs of patients and to the organizational needs of the system (goal-management, documentation requirements, budget-cuts), and in many ways they carry the emotional burden of a complex, rigid healthcare system.

Organizational needs based on NPM-principles cannot be justified by patients’ health or wellbeing, which is the fundamental reason for the healthcare systems existence. Providing high-quality nursing can be demanding within the systems framework of predefined decisions that nurses are legally obligated to fulfill [36]. Studies show that nurses use relatively much time on reporting and documentation, and nurses striving to provide comprehensive care while simultaneously meeting management’s requirements may lower the quality of patient care and of administrative tasks [33]. Nurses’ abilities may diminish in maintaining NPRs and in assessing, understanding and meeting immediate and long-term needs. As a result, fundamental patient needs remain unfulfilled, leaving patients particularly vulnerable to dealing with unmet emotional, psychosocial, physical, and educational needs [66, 67]. Organizational and financial constraints seem to prevent nurses from doing what they believe is right, which may cause nurses to experience organizational moral distress [68].

Findings indicate that, rather than rigidly abiding by regulations that the organization imposes, nurses use individual strategies to create the flexibility to adjust care according to patients’ needs, thereby demonstrating their loyalty to patients, and their disobedience towards management. Providing services beyond what is assigned in the decisions has previously been referred to as ‘hidden services’ [69]. Patients demonstrate their devotion to nurses by trying to ease their work and by accepting that nurses cannot help with all their needs. Many of the actions of nurses and patients seem to be based on their experiences of the value of unmeasurable tasks belonging to the lifeworld, such as having conversations, establishing caring NPRs, being present, etc. [18].

Nurses’ disobedience may also be attempts to maintain their professional autonomy, and their disobedient practices appear to be accepted by patients, colleagues, and nurse-leaders. Choices to game the system may be a result of misalignment between the principles, values, and priorities of nurses/patients and management, i.e. conflicts between the lifeworld and the system [64]. However, the overall policy goals of Norwegian municipal healthcare broadly coincide with nursing goals, and revolve around enhancing health and caring for fundamental needs, including social interaction, companionship, self-care, etc. [3, 4, 70]. Therefore, it is not necessarily that different goals create the misalignment, but rather the management processes towards meeting those goals. This suggests that current management processes need to be altered in order to better facilitate for policy goals to be met. Even though healthcare authorities are concerned with quality and value-oriented objectives, these are difficult to measure; instead, nursing work is measured in quantifiable goals, such as number of minutes or patients [71]. While gaming the system undermines management, it simultaneously safeguards the municipal healthcare system’s overall policy goals. Thus, we would recommend management processes that involve less management, control and measurement of nurses’ tasks, and grant nurses more authority and freedom to evaluate how to spend their time in caring and health-enhancing purposes.

Although the intentions behind gaming the system may be honorable, the practice can also have unfortunate consequences. Administrative tasks, documentation, and correspondence with other patients by telephone are often performed while nurses work with patients, which may violate professional confidentiality. When nurses choose to not abide by predefined tasks, the allocation of resources are based on the individual nurse’s evaluations which may lead to inequality and unfairness in providing care, as more demanding patients and families receive higher priority [32]. Consequently, the weakest patients may suffer. As findings show, many patients are grateful for any help they receive and place few demands on nurses; these patients are vulnerable to unfair resource allocation. Furthermore, if nurses spend their energy and focus on manipulating the system rather than caring for the patients, it may cause suffering for patients and burnout in nurses [48, 72]. Patients may also be at risk for burnout if they constantly adjust their behavior to ease nurses’ work. Many patients express that it is important to be able to remain in their home, and they might feel pressured to adjust their behavior to ensure they are not placed in institutions. One can ask if it is morally justifiable to place such demands on patients.

Positive outcomes for nurses gaming the system may be that they are able to perform their profession in somewhat compliance with their own professional ideals, and that by neglecting to document their true actions, they do not have to justify their choices to their leaders. When nurses and their leaders have conflicting perceptions of what constitutes the patient’s best interest, nurses may be less likely to engage in organizational dialogues, and a form of indifference may consequently arise. Nurse-leaders advocate for both the patient and the nurse in the financial resource-allocation discussion in healthcare organizations. Nurse-leaders are part of the home-care culture, and they possess the key to creating the best ethical-caring culture [73, 74]. Choices to game the system seem to be influenced by the ward culture and leaders. Since many leaders are nurses, they likely share nurses’ perceptions, and leaders may quietly condone nurses’ disobedience, thus allowing the gaming to continue.

When nurses multitask, manipulate timers, work through breaks, avoid logging overtime, or rush/skip tasks, it creates an appearance that the allocated decision-time is sufficient, and the challenges remain hidden [69]. Through nurses’ and patients’ compensatory actions, the unfortunate consequences of NPM-organization are concealed, and NPM appears more successful than it really is. Policy makers and leaders may assume that nurses will continue to provide comprehensive care, despite understaffing and increased workload [6]. Studies indicate that understaffing is a way of forcing nurses to make rationing choices at the bedside, as well as high prevalence of missed care responsibilities like preventative care, disadvantaged groups, administration etc. [6, 75]. A worrying finding in this study is nurses’ feelings of wanting to give up and surrender to the system. This may indicate that NPM’s prioritization of goals and values may over time wear down nurses’ compensatory actions. The problem is: how long nurses can retain their professional identity if the core content is counteracted by organizational boundaries? Thus, we might not yet have seen the full negative effects of NPM in home care.

Effective management of nursing resources is essential for a sustainable society. Since nursing time is a limited commodity, nurses may have to prioritize more medical, technical, and administrative tasks in the future, with potential negative consequences on NPRs and individualized care. Organizational frameworks can help structure and formalize allocation of scarce resources, but paragraphs and worklists cannot replace empathy and common sense. Nurses become unable to make independent assessments, tailor care, and establish relationships if systems are too rigid. Although gaming the system might have unfortunate consequences, the alternative (following all the rules without deviating) shifts the focus away from patients and undermines the nursing profession.

Findings show that the amount of time nurses spend on administrative work, documentation, driving, interdisciplinary work, and unforeseen events is greatly underestimated by policy makers, which indicates the latter as being removed from reality and only having selective knowledge of what nursing actually entails and the conditions under which home-care nursing is provided. We advocate that nurses should be involved as key players in determining how services and resources should be assigned and allocated. Standardizing services and actions in home care might seem effective and profitable, but the NPM-organization may be neither economically sound nor supportive of high-quality nursing [10]. Patients do not necessarily receive the care and assistance they truly need, and the health-enhancing potential of unmeasurable tasks, such as conversations, go underestimated.

### Methodological considerations

Findings of this study may vary in degree of validity and transferability to other settings, as focused ethnographic studies are contextual by nature [53, 76]. Some findings, such as misalignments between the values and priorities of nurses/patients and management, may have high transferability to other parts of the healthcare system, both in Norway and internationally. Other findings, such as disobedient behavior, may be transferable to contexts with similar organizational systems, and some findings may have less validity outside the examined fields.

All data was collected by the researcher (MS), who as a nurse had a pre-understanding of the context of the current healthcare system which may have influenced observations and the research-process. To address this, the researcher frequently asked clarifying questions to gain deeper understanding, and all authors reflected critically on the findings. Rigor in this study rests on the credibility and dependability of the findings and on the auditability of the work. We attempted to be as reflexive as possible, seeking to describe carefully the context, data collection, and analysis [77, 78], while also revealing the richness of the data and thick descriptions [79]. Triangulating sources (observations and nurse/patient interviews) may minimize limitations, as they revealed different perspectives on the issues of the study aim. Verbatim quotes and field notes illustrate and validate the themes. The article complies with the COREQ guidelines [80].

Despite limitations, this study offers new knowledge that is closely related to nurses’ and patients’ reality, knowledge that nurses need to provide the care needed and wished for by patients in home care. In order to change the system, nurses need to collaborate and engage in dialogue with their leaders to act on behalf of the patients in home care [73].

## Conclusions

This focused ethnographic study identifies cultural patterns in the ways nurses and patients relate to and deal with organizational systems in Norwegian home care. Findings show that patients adjust their behavior and lower their demands in order to ease the burden on home care nurses, while nurses choose to manipulate the organizational system to perform tasks according to their own professional assessments. Their actions are based on assumptions regarding which aspects of nursing are most important to enhance patients’ health and ensure wellbeing - viewing NPRs, individualized care and caring, as essential, but perceiving them to be devalued in the NPM-organization. Our findings indicate that, in many ways, the organizational system hampers provision of high-quality nursing, and that comprehensive care is provided despite, not because of, the system. Nurses and patients compensation of flaws in the organizational system may unintentionally support the same system, as their actions conceal problematic sides and negative consequences of NPM-organization, thus making NPM appear more successful than it actually is.

It is important to understand the meanings behind these cultural practices and beliefs in order to evaluate the current organizational system and to develop effective, professional and high-quality healthcare services. Patients’ needs must be addressed properly in policy documents and guidelines in order to ensure fair allocation based on the complexity of patients’ needs. Home-care nurses and patients should be heard regarding which needs and tasks to prioritize, and they should be assigned a more significant role in determining how recourses are allocated. Further discussion is also needed on the role, scope, and responsibilities of home care nurses, focusing on the wide range of ethical choices nurses have to make.

## Abbreviations

NPM: New Public Management
NPR: Nurse-patient relationship

## References

[1] Vabø M (2012). Norwegian home care in transition - heading for accountability, off-loading responsibilities. Health and Social Care in the community.

[2] Greve C, Lægreid P, Rykkja L, Greve C, Lægreid P, Rykkja L (2016). Introduction: the Nordic model in transition. Nordic administrative reforms: lessons for public management.

[3] Kvalitetsforskrift for pleie- og omsorgstjenestene [Quality regulations for nursing and care services] (2003). Forskrift om kvalitet i pleie- og omsorgstjenestene for tjenesteyting etter lov av 19. november 1982 nr. 66 om helsetjenesten i kommunene og etter lov av 13. desember 1991 nr. 81 om sosiale tjenester m.v.

[4] Helse- og omsorgstjenesteloven [Healthcare service act] (2011). Lov om kommunale helse- og omsorgstjenester m.m. av 24 Juni 2011 nr. 30 [the act on municipal healthcare services].

[5] OECD. Health at a Glance 2017: OECD Indicatiors. Paris: OECD Publishing; 2017.

[6] Scott AP, Harvey C, Felzmann H, Suhonen R, Habermann M, Halvorsen K, et al. Resource allocation and rationing in nursing care: a discussion paper. Nursing Ethics. 2018. 10.1177/0969733018759831. 10.1177/0969733018759831 PMC6681425 29607703

[7] Vabø M (2007). Organisering for velferd: Hjemmetjenesten i en styringsideologisk brytningstid [Organization for welfare: Home care service in management-ideological upheaval].

[8] Christensen T, Lægreid P (2001). New public management: the effects of Contractualism and devolution on political control. Public Manag Rev.

[9] Wollscheid S, Eriksen J, Hallvik J (2013). Undermining the rules in home care services for the elderly in Norway: flexibility and cooperation. Scand J Caring Sci.

[10] Hood C, Dixon RA (2015). Government that worked better and cost less? Evaluating three decades of reform and change in UK central government.

[11] Nortvedt P, Pedersen R, Grothe K, Nordhaug M, Kirkevold M, Slettebo A (2008). Clinical prioritisations of healthcare for the aged - professional roles. J Med Ethics.

[12] Austin WJ (2011). The incommensurability of nursing as a practice and the customer service model: an evolutionary threat to the discipline. Nurs Philos.

[13] Tønnessen S, Nortvedt P, Førde R (2011). Rationing home-based nursing care: professional ethical implications. Nurs Ethics.

[14] Dale B, Sævareid HI, Kirkevold M, Söderhamn O (2011). Older home-living patients perceptions of received home nursing and family care. Nordisk sygeplejeforskning.

[15] Haukelien H, Vike H, Vardheim I. Samhandlingsreformens konsekvenser i de kommunale helse- og omsorgstjenestene. Sykepleieres erfaringer [Concequences of the Coordination Reform in Municipal Healthcare services]. Bø i Telemark; 2015. Report no.: TF-rapport nr. 362.

[16] Uhrenfeldt L, Hall EO (2015). Job satisfaction as a matter of time, team, and trust: a qualitative study of hospital nurses’ experiences. J Nurs Educ Pract.

[17] Willis E, Toffoli L, Henderson J, Couzner L, Hamilton P, Verrall C (2016). Rounding, work intensification and new public management. Nurs Inq.

[18] Strandås M, Wackerhausen S, Bondas T (2019). The nurse-patient relationship in the new public management era, in public home care: a focused ethnography. J Adv Nurs.

[19] Leininger MM, McFarland MR (2002). Transcultural nursing: concepts, theories and practices.

[20] Martinsen K (2005). Samtalen, skjønnet og evidensen [Conversation, professional judgement and evidence]. Oslo: Akribe.

[21] Killett A, Burns D, Kelly F, Brooker D, Bowes A, La Fontaine J, et al. Digging deep: how organisational culture affects care home residents' experiences. Ageing and Society. 2016;36(1):160–88.

[22] Samhandlingsreformen [Coordination Reform] (2009). Samhandlingsreformen - Rett behandling – på rett sted – til rett tid [the coordination reform - Rigth treatment - at the right place - at the right time].

[23] Mørk E, Beyrer S, Haugstveit FV, Sundby B, Karlsen HT. Kommunale helse- og omsorgstjenester 2017 - Statistikk om tjenester og tjenestemottakere [Municipal healthcare services 2017 - Statistics on services and service recipients]. Oslo; 2018. Report No.: 2018/26.

[24] Nylenna M (2014). Helsetjenesten i Norge. Et overblikk [Healthcare services in Norway. An overview].

[25] OECD (2011). Health reform.

[26] Statistisk Sentralbyrå [Statistics Norway]. Dette er Norge 2015 - Hva tallene forteller [This is Norway - What the numbers say]. Oslo; 2015. Report No.: ISBN 978-82-537-9171-5.

[27] Markel-Reid M, Weir R, Browne G, Gafni A, Henderson S (2006). Health promotion for frail older home care clients. J Adv Nurs.

[28] Helse- og omsorgsdepartementet [Ministry of health and care]. Meld. St. 29 (2012–2013) Morgendagens omsorg. [Care of tomorrow] Oslo: Helse- og omsorgsdepartementet [Ministry of health and care]; 2013 [Available from: https://www.regjeringen.no/contentassets/34c8183cc5cd43e2bd341e34e326dbd8/no/pdfs/stm201220130029000dddpdfs.pdf.

[29] Sæterstrand TM, Holm SG, Brinchmann BS (2015). Hjemmesykepleiepraksis. Hvordan ny organisering av helsetjenesten påvirker sykepleiepraksis [Home care nursing practice. How the new organization of the healthcare services affects nursing practice.]. Klinisk Sygepleje.

[30] Statistisk Sentralbyrå [Statistics Norway]. Folkemengde og befolkningsendringar [Population and population changes] 2018 [Available from: https://www.ssb.no/befolkning/statistikker/folkemengde/kvartal.

[31] Vabø M (2009). Home care in transition: the complex dynamic of competing drivers of change in Norway. J Health Organ Manag.

[32] Papastavrou E, Andreou P, Efstathiou G (2014). Rationing of nursing care and nurse–patient outcomes: a systematic review of quantitative studies. Int J Health Plann Manag.

[33] Gjertsen H, Solvoll G, Gjernes T. Tidsbruk og byråkrati i pleie- og omsorgstjenestene: En studie av omfang, nytte og kostnader ved rapporterings- og dokumentasjonsarbeid i kommunale pleie- og omsorgstjenester [Time-use and bureaucracy in healthcare services: A study of the extent, utility and costs of reporting- and documentation work in municipal healthcare services]. Bodø; 2012. Report No.: NF-rapport nr. 12/2012.

[34] Gautun H, Syse A. Samhandlingsreformen - Hvordan tar de kommunale helse- og omsorgstjenestene i mot det økte antallet pasienter som skrives ut fra sykehuset? [The Coordination reform - How do municipal healthcare services deal with the increased number of patients discharged from hospitals?]. Oslo; 2013. Report No.: NOVA Rapport 8/13.

[35] Forskrift om egenandel for kommunale helse- og omsorgstjenester [Regulations on deductibles for municipal healthcare services]. Forskrift om egenandel for kommunale helse- og omsorgstjenester av 16. Desember 2011 nr. 1349 2011 [Available from: https://lovdata.no/dokument/SF/forskrift/2011-12-16-1349.

[36] Forvaltningsloven. Lov om behandlingsmåten i forvaltningssaker av 10 Februar 1967 [Act relating to procedure in cases concerning the Public Adm]. 1967 [Available from: https://lovdata.no/dokument/NLE/lov/1967-02-10.

[37] Pasient- og brukerrettighetsloven. Lov om pasient- og brukerrettigheter av 02. Juli 1999 nr. 63 [Act on patient and user rights]. 1999 [Available from: https://lovdata.no/dokument/NL/lov/1999-07-02-63?q=pasientrettighetsloven.

[38] Helsedirektoratet [Directorate of Health]. IPLOS Veileder for personell i kommunale helse- og omsorgstjenester [Guide for personell in municipal healthcare services] Oslo: Helsedirektoratet [Directorate of Health]; 2015 [Available from: https://helsedirektoratet.no/Lists/Publikasjoner/Attachments/436/Veileder%20for%20personell%20i%20kommunale%20helse-%20og%20omsorgstjenester.pdf.

[39] Holm SG, Mathisen TA, Sæterstrand TM, Brinchmann BS. Allocation of home care services by municipalities in Norway : a document analysis. BMC Health Serv Res. 2017;17(673). 10.1186/s12913-017-2623-3 PMC5610450 28938892

[40] Helsedirektoratet [Directorate of Health] (2016). Veileder for saksbehandling - Tjenester etter helse- og omsorgstjenesteloven §§ 3-2 første ledd nr. 6, 3–6 og 3–8 [guide for case processing].

[41] Holm SG, Angelsen RO (2014). A descriptive retrospective study of time consumption in home care services: how do employees use their working time?. BMC Health Serv Res.

[42] Dinç L, Gastmans C (2013). Trust in nurse–patient relationships. Nurs Ethics.

[43] Uhrenfeldt L, Sørensen EE, Bahnsen IB, Pedersen P (2018). The centrality of the nurse–patient relationship: a Scandinavian perspective. J Clin Nurs.

[44] Arman M, Ranheim A, Rydenlund K, Rytterström P, Rehnsfeldt A (2015). The Nordic tradition of caring science: the works of three theorists. Nurs Sci Q.

[45] Strandås M, Bondas T (2018). The nurse-patient relationship as a story of health enhancement in community care: a meta-ethnography. J Adv Nurs.

[46] Kihlgren A, Blomberg K, James I (2015). A reciprocal relationship - an opportunity and a solution for a meaningful daily life in home care - the older person’s perspective. Clinical nursing. Studies.

[47] Eriksson K (2002). Caring science in a new key. Nurs Sci Q.

[48] Fläckman B, Skovdahl K, Fagerberg I, Kihlgren M, Kihlgren A (2015). Consequences of working in eldercare during organizational changes and cut backs while education and clinical supervision was provided: a mixed methods study. Open J Nurs.

[49] Dadich A, Collier A, Hodgins M, Crawford G (2018). Using positive organizational scholarship in healthcare and video reflexive ethnography to examine positive deviance to new public Management in Healthcare. Qual Health Res.

[50] Berggren I, Carlström E (2010). Decision making within a community provider organization. Br J Commun Nurs.

[51] Jensen SM, Villadsen K, Gubrium JF, Andreassen TA, Solvang PK (2016). "civil disobedience" and conflicting rationalities in elderly care. Reimagining the human service relationship.

[52] Roper JM, Shapira J (2000). Ethnography in nursing research.

[53] Higginbottom GMA, Pillay JJ, Boadu NY (2013). Guidance on performing focused ethnographies with an emphasis on healthcare research. Qual Rep.

[54] Morse JM, Barrett M, Mayan M, Olson K, Spiers J (2002). Verification strategies for establishing reliability and validity in qualitative research. Int J Qual Methods.

[55] Spradley J (1980). Participant observation.

[56] Schein EH (2010). Organizational culture and leadership.

[57] Cruz EV, Higginbottom G (2013). The use of focused ethnography in nursing research. Nurse Researcher.

[58] Polit D, Beck C (2012). Nursing research - generating and assessing evidence for nursing practice.

[59] Fangen K (2010). Deltagende observasjon [Participant observation]. 2. utg. ed.

[60] Kvale S, Brinkmann S. Det kvalitative forskningsintervju [The Qual Res interview]. Oslo: Gyldendal akademisk; 2009. 344 s. : ill. p.

[61] QRS International. NVivo qualitative data analysis Softeware. 11 ed2017.

[62] World Medical Association. Declaration of Helsinki - Ethical Principles for Medical Research Involving Human Subjects 2013 [Available from: https://www.wma.net/policies-post/wma-declaration-of-helsinki-ethical-principles-for-medical-research-involving-human-subjects/. 10.1001/jama.2013.281053 24141714

[63] Willis E, Carryer J, Harvey C, Pearson M, Henderson J (2017). Austerity, new public management and missed nursing care in Australia and New Zealand. J Adv Nurs.

[64] Habermas J (1999). Kommunikasjon, handling, moral og rett [the theory of Communicatove action].

[65] Fläckman B, Fagerberg I, Häggström E, Kihlgren A, Kihlgren M (2007). Despite shattered expectations a willingness to care for elders remains with education and clinical supervision. Scand J Caring Sci.

[66] Dahlberg K (2002). Vårdlidande - det onödige lidande [Suffering from care - The unnecessary suffering]. Vård i Norden.

[67] Eriksson K (2010). Det lidende menneske [the suffering human being]. 2. Udg. Ed..

[68] Mills AE, Werhane PH. Organizational Moral Distress. In: Cooper CL, editor. Wiley Encyclopedia of Management2015.

[69] Kirchhoff J (2010). De skjulte tjenestene : Om uønsket atferd i offentlige organisasjoner [The hidden services: About unwanted behavior in public organizations].

[70] Parse RR (1998). The human becoming School of Thought.

[71] Jørgensen SL. Anerkendende ledelse under budgetpres [Recognizing management under budget pressure] Økonomistyring og Informatik [Economic management and Informatics] 2014;29(3):245–87.

[72] Glasberg AL, Eriksson S, Norberg A (2007). Burnout and ‘stress of conscience’ among healthcare personnel. J Adv Nurs.

[73] Bondas T (2003). Caritative leadership: ministering to the patients. Nurs Adm Q.

[74] Bondas T (2009). Preparing the air for nursing care: a grounded theory study of first line nurse managers. J Res Nurs.

[75] Phelan A, McCarthy S, Adams E (2018). Examining missed care in community nursing: a cross section survey design. J Adv Nurs.

[76] Knoblauch H (2005). Focused ethnography. Forum Qualitative Social Research.

[77] Lincoln YS, Guba EG (1985). Naturalistic Inquiry.

[78] Sandelowski M (1986). The problem of rigor in qualitative research. Adv Nurs Sci.

[79] Geertz C (1993). The interpretation of cultures : selected essays.

[80] Tong A, Sainsbury P, Craig J (2007). Consolidated criteria for reporting qualitative research (COREQ): a 32-item checklist for interviews and focus groups. Int J Qual Health Care.

